# P06-14 Development of a standardised evaluation framework (SEF) for physical activity interventions: Ensuring usability and usefulness

**DOI:** 10.1093/eurpub/ckac095.099

**Published:** 2022-08-29

**Authors:** Joey Murphy

**Affiliations:** Centre for Exercise, Nutrition and Health Sciences, School for Policy Studies, Bristol

**Keywords:** Evaluation, standardised, tool, usability, pragmatic

## Abstract

**Introduction:**

With a range of interventions available for promoting physical activity (PA) and only limited resources, it has become imperative to identify those that are both effective and feasible for real world application. A number of evaluation frameworks have been developed but often fail to be widely implemented due to the level of information required and time needed to complete. The purpose of this study is to develop a standardised evaluation framework (SEF) for PA interventions that is usable in practice but also collects information that enables evidence based decision making among key knowledge users.

**Methods:**

Development of the framework was guided by Nutbeam and Bauman's evaluation cycle, encompassing elements of formative, process, impact and outcome evaluation. The SEF was developed through four stages involving a 1) review of the literature, 2) feedback from key stakeholders, 3) national consultation, and 4) focus groups with a practitioner advisory group. Elements of the Technology Acceptance Model were used to assess the perceived usefulness and usability of the SEF by key stakeholders.††

**Results:**

Twelve relevant evaluation frameworks specific to PA interventions were identified through the literature review. Members of the project team (N = 3) identified commonalities across these frameworks, including general characteristics (N = 12), formative evaluation aspects (N = 5), process evaluation aspects (N = 15), impact evaluation aspects (N = 7) and outcome evaluation aspects (N = 3). Feedback across four stages, including consultations, two focus groups and an online survey provided feedback for creating a more usable and useful SEF. The current framework includes a minimum set of questions (i.e. monitoring template) for coordinators (N = 27) and participants (N = 9) with additional measures available for an in-depth evaluation where necessary.

**Conclusion:**

I-PARC has seen the creation of a SEF that is moving towards a more usable approach for intervention evaluation in practice. The I-PARC SEF has the potential to be a usable resource to assess current PA interventions and provide knowledge regarding the potential scale up, adaptions or cessation of current practices. Feedback has also suggested a need for an online platform to collect the relevant information, capacity building resources and a support network to help with the use of the I-PARC SEF.

